# Motif-directed redesign of enzyme specificity

**DOI:** 10.1002/pro.2417

**Published:** 2014-02-04

**Authors:** Benjamin Borgo, James J Havranek

**Affiliations:** 1 Program in Computational and Systems Biology, Washington University in St. LouisSt. Louis, Missouri, 63110; 2 Department of Genetics, Washington University in St. LouisSt. Louis, MO, 63110

**Keywords:** computational protein design, mutagenesis, molecular specificity, enzyme design

## Abstract

Computational protein design relies on several approximations, including the use of fixed backbones and rotamers, to reduce protein design to a computationally tractable problem. However, allowing backbone and off-rotamer flexibility leads to more accurate designs and greater conformational diversity. Exhaustive sampling of this additional conformational space is challenging, and often impossible. Here, we report a computational method that utilizes a preselected library of native interactions to direct backbone flexibility to accommodate placement of these functional contacts. Using these native interaction modules, termed motifs, improves the likelihood that the interaction can be realized, provided that suitable backbone perturbations can be identified. Furthermore, it allows a directed search of the conformational space, reducing the sampling needed to find low energy conformations. We implemented the motif-based design algorithm in Rosetta, and tested the efficacy of this method by redesigning the substrate specificity of methionine aminopeptidase. In summary, native enzymes have evolved to catalyze a wide range of chemical reactions with extraordinary specificity. Computational enzyme design seeks to generate novel chemical activities by altering the target substrates of these existing enzymes. We have implemented a novel approach to redesign the specificity of an enzyme and demonstrated its effectiveness on a model system.

## Introduction

Computational protein design has advanced rapidly over the past decade. Despite many impressive successes,^1^–^9^ generating novel, functional proteins with activity levels similar to natural proteins remains challenging. Limitations in scoring functions, structural representation, and search strategies provide ample opportunity for improvement. A common strategy for circumventing these limitations is to incorporate structural building blocks from experimentally determined structures. For example, computational protein design typically involves the combinatorial selection of experimentally observed amino acid conformations (rotamers) that optimize some scoring function when arranged on the backbone of a native protein.^4^,^10^–^13^ Similarly, structure prediction algorithms often rely upon libraries of backbone fragments culled from the protein databank to reduce the conformational space that must be sampled when assembling structural models of proteins.^14^–^18^ This strategy involves a trade-off: native structural building blocks ensure that our models contain plausible interactions, but bias us towards what has already been observed. This can be particularly limiting for protein design, which usually seeks to realize a novel function or specificity.

Two strategies for leveraging native protein structures present themselves in the context of the design of protein function. At the macromolecular level, we can identify proteins that carry out related functions as starting templates and attempt to preserve aspects of this function while redesigning other residues to accommodate desired changes. At the atomic level, we can identify specific interactions involving residues in unrelated proteins that may prove useful in achieving the change in function or specificity that we require to move from a starting template to a novel molecule. We can direct the choice of mutations required to repurpose the native template by focusing on recreating previously observed interactions from other structural contexts. Using previously observed functional interactions is likely to increase the odds of success for a given protein engineering goal.

The transplantation of atomistic interactions onto a design template is challenging because the backbone conformation of the template is unlikely to present an optimal geometry to reproduce the interactions found in the native context. Introducing modest backbone flexibility is likely to accommodate a large number of functional interactions, but it is not possible to know *a priori* how the backbone should be deformed. We previously described a computational algorithm for addressing this problem in the context of protein-DNA interactions.^19^ We identify a set of previously observed functional interactions (called motifs) and attempt to transplant them onto our design template. A motif can be successfully incorporated if modest movement of the design template backbone accommodates placement of the motif's functional amino acid. A previous computational approach to enzyme redesign utilized flexible backbones^20^; however, this did not rely on library of putative functional interactions and required explicit selection of mutatable positions.

In this report, we extend the method introduced in Ref. 19. for selecting motifs from a set of native interactions to confer a change in the specificity of an enzyme. Starting with a library of potentially functional motifs, we implement an algorithm that utilizes iterative cycles of backbone relaxation and motif placement followed by the redesign of additional supporting mutations. The extended method is general in the sense that it can be used to design for any target for which a comprehensive motif-library can be gathered from databases or constructed from computations. We describe this approach in detail, and demonstrate its effectiveness by altering the specificity and activity of methionine aminopeptidase. Our results show that in this system, the transfer of residue-level functional interactions can alter substrate specificity while preserving existing catalytic activity.

## Results

### Computational redesign of specificity with backbone flexibility

**Figure 1:**
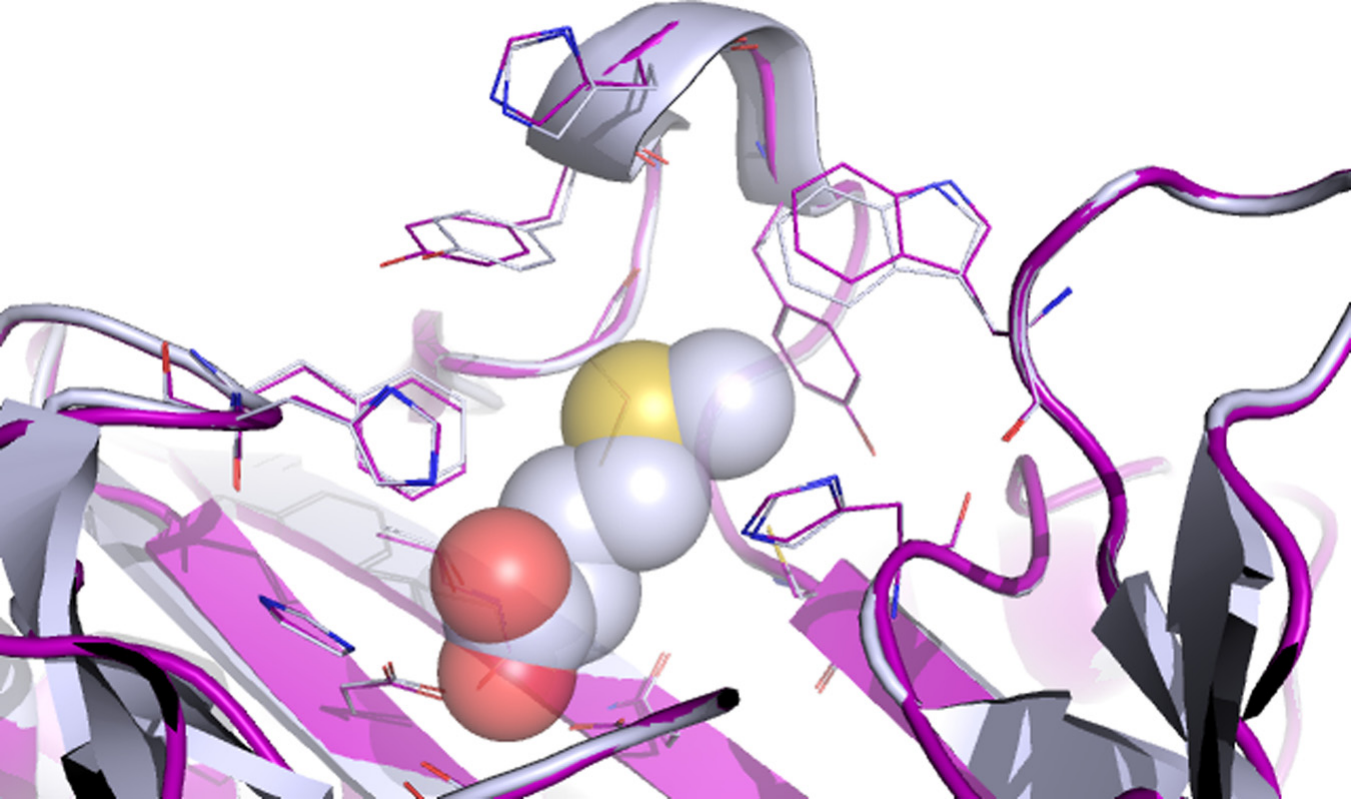
Holo-eMAP (PDB ID *2MAT*,^22^ white) shows minimal conformational changes (0.114 Å RMSD) when bound with methionine (PDB ID *1C21*,^24^ purple). The residues that contact the substrate are shown in a stick representation.

**Figure 2:**
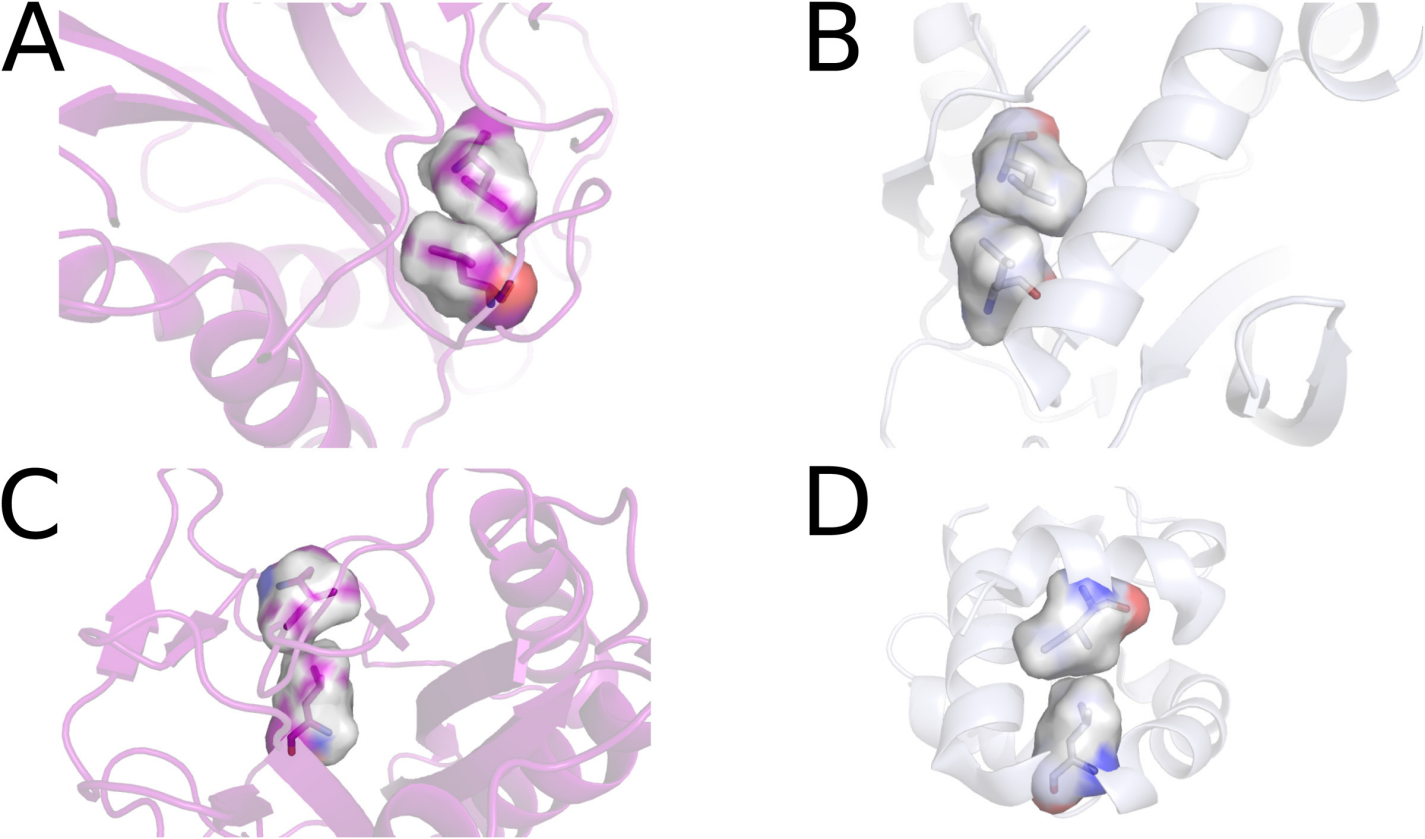
Motif donors. The placed motifs are shown here in their final position in eLAP (A,C) and their native background (B,D). The native contact orientation is maintained throughout the design process by constraining the three atoms that define the motif coordinate system. Backbone atoms are allowed to move in discrete “inverse-rotameric” conformations to graft the motif into its acceptor position.

Methionine aminopeptidase from *E. coli* (eMAP) is an essential metallo-aminopeptidase responsible for post-translational removal of N-terminal methionine from proteins. As shown in Figure 2, the N-terminal methionine is directly contacted by two loop regions surrounding the active site. A number of residues required for catalysis have been identified by mutagenesis,^21^–^23^ while those involved in substrate recognition are less well-studied but can be inferred from the crystal structures. Comparing the apo and holo forms of the protein (purple and white, respectively, in Figure 1) reveals a relatively rigid binding pocket that shifts only slightly during substrate recognition. This “lock-and-key” binding site is amenable to specificity redesign since no large structural rearrangements appear to be required to accommodate the substrate. We therefore sought to switch the specificity for N-terminal methionine to specificity for N-terminal leucine using motif-based design. This is a relatively stringent test for specificity redesign since leucine and methionine both have similar hydrophobic properties and are comparable in size.^25^

### Flexible backbone design can be directed by interaction motifs

A loop region of the protein recognizes the sidechain of the methionine substrate. We anticipated that the loop region would need to rearrange to recognize a leucine sidechain. Because optimal loop conformations for recognition cannot be determined in the absence of side chain-side chain interactions, we employed motif-directed design. In this approach, a library of previously observed amino acid-amino acid contacts is collected from a set of experimentally determined structures.

Each motif in the library is used to place a free, interacting amino acid in the appropriate location to realize the interaction with the desired leucine substrate. An inverse rotamer library^19^ is used to sample the side chain degrees of freedom of the introduced amino acid. This yields a set of virtual amino acids poised to reproduce previously observed interactions with leucine, each with an enumerated set of backbone locations that could give rise to the interaction. A computational procedure is employed to search for interactions with the substrate that can easily be incorporated into the preexisting backbone of eMAP with a limited amount of conformation rearrangement.

**Figure 3:**
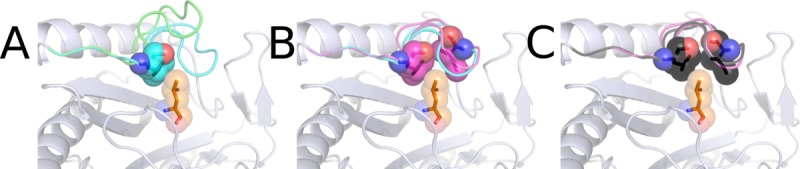
eLAP design. (A) Initial placement of the LL motif (cyan, sphere representation) pulls the remodeled loop (cyan, cartoon) inwards towards the substrate (orange). This step repositions the loop from its native conformation (green). (B) A second motif, the LI motif (purple, right) is placed adjacent to the substrate, and the loop is again remodeled (purple, cartoon) to accommodate the new motif. The LL motif is constrained in this step. (C) Residues surrounding both motifs in the loop region (black) are mutated to support the dual-motif placement, and relaxed in 10 iterations. Resulting loop movement is minimal, and the native interactions are maintained during remodeling.

**Figure 4:**
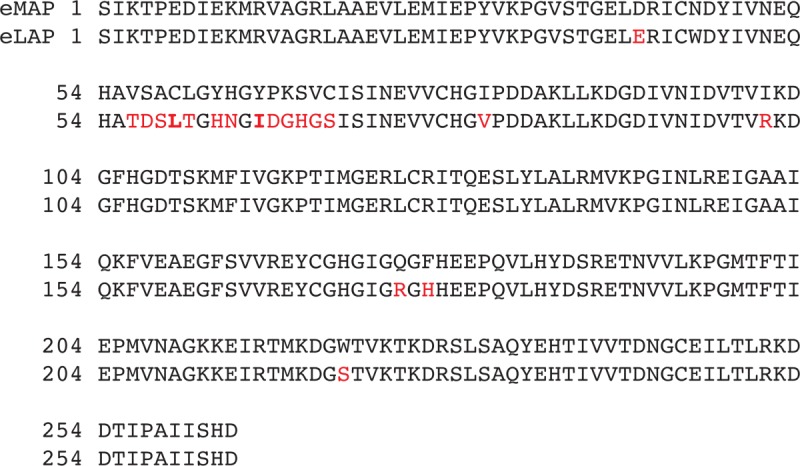
Alignment of methionine aminopeptidase with the final design for a leucine aminopeptidase. The flexible loop region encompasses residues 56–70 with the two motif placements shown in bright red. Additional mutations made to accommodate a greater range of loop conformations are shown in bold letters.

Using this approach, we identified residue-level interactions that could be accommodated by the eMAP backbone with conformational flexibility. The first is an interaction between two leucine residues [Fig. 2(A,B)] taken from GTP cyclohydrolase II (positions 47A and 18A, PDB code: *2BZ1*^26^). The second is between residues Leu428A and Ile378A [Fig. 2(C,D)] from estrogen receptor α (PDB code: *2IOK*^27^). To maximize flexibility during the search for compatible backbone-motif matches, we changed residues within the loop to alanine, with the exception of glycine and proline amino acids, which were unchanged. Following the iterative incorporation of the two interaction motifs, nonmotif residues within the loop were redesigned using the RosettaDesign^28^ program (Fig. 3). This process was repeated 10 times, with backbone relaxation performed between each sequence redesign calculation.^29^,^30^ The resulting protein is denoted eLAP, and differs from eMAP at 19 positions (Fig. 4).

### Inactive eMAP exhibits binding specificity for N-terminal methionine peptides

**Figure 5:**
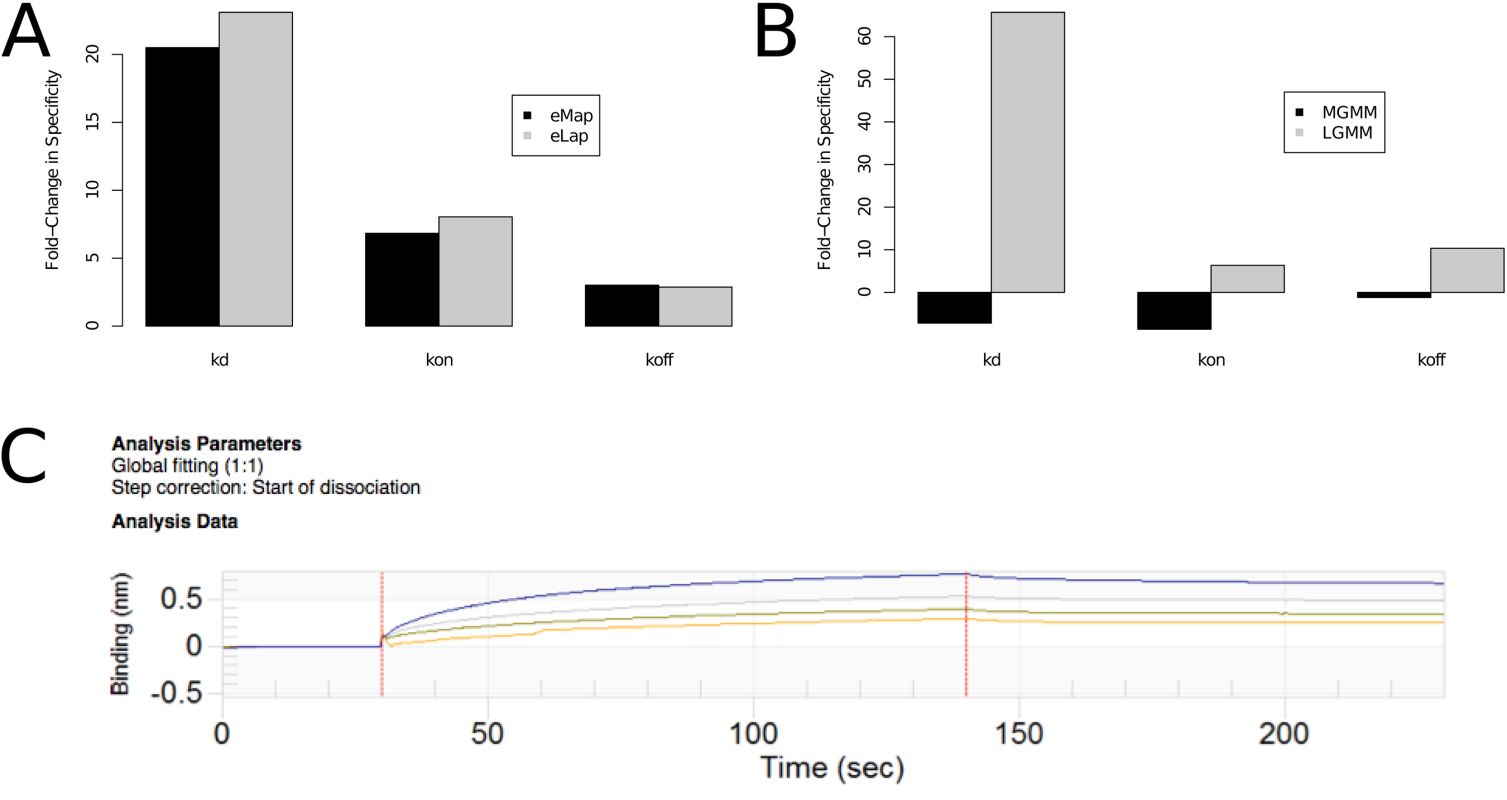
Changes in specificity in designed eLAP. On, off and dissociation rates for eMAP and eLAP show similar specificities (∼20-fold affinity preference) for their target substrates (A), indicating that motif-based design successfully changes the specificity of eMAP. A comparison of binding to each substrate (B), shows that the primary increase in affinity is for the positive design state (ie eLAP for leucine) rather than against methionine. A raw sensogram used to derive the specificity comparisons is shown in (C).

In the presence of a metal chelator, we found that eMAP binds N-terminal methionine noncatalytically. We first conducted bio-layer interferometry (BLI) experiments to measure the affinity of native eMAP for an N-terminal methionine peptide ligand. The results indicate that methionine recognition by eMAP in the absence of catalytic activity is a fairly weak interaction, with a dissociation constant of 2.65 μ*M*. Next, we assayed the binding activity of eMAP against an N-terminal leucine peptide ligand. The measured dissociation constant was 54.2 μ*M* (Table I). Thus, eMAP exhibits a >20-fold specificity preference for N-terminal methionine over leucine (Fig. 5, Table I).

**Table I:** Binding parameters of native and designed proteins

Protein/peptide	*k*_on_ (M^−1^ s^−1^)	*k*_off_ (s^−1^)	*K*_D_ (µ*M*)
Emap/Met	1.85 × 10^3^	4.89 × 10^−3^	2.65
Emap/Leu	2.71 × 10^2^	1.47 × 10^−2^	54.2
Elap/Met	2.14 × 10^2^	4.08 × 10^−3^	19.1
Elap/Leu	1.72 × 10^3^	1.42 × 10^−3^	0.826

### Inactive eLAP exhibits altered specificity profiles for N-terminal methionine and leucine

We next tested whether our designed eLAP protein possessed altered specificity for the N-terminal methionine and leucine peptides. Ideally, a specificity “swap” would result not only in a change of relative binding preferences relative to eMAP (such a mutant may still prefer methionine, but by a smaller amount), but in an absolute preference for leucine over methionine. We first measured the affinity of eLAP for the N-terminal leucine peptide and found detectable binding with a dissociation constant of 0.83 μ*M*. This is slightly better than the affinity of eMAP for the methioinine peptide. We then attempted to confirm the specificity swap by measuring the eLAP affinity for methionine. The resulting dissociation constant (*K*_d_ = 19.08 μ*M*) is more than an order of magnitude higher than eLAP for leucine. Thus, eLAP exhibits a 20-fold preference for leucine over methionine (Fig. 5), verifying that the mutant's affinity profile is opposite that of eMAP.

### Active eLAP exhibits altered activity for N-terminal methionine and leucine substrates

**Figure 6:**
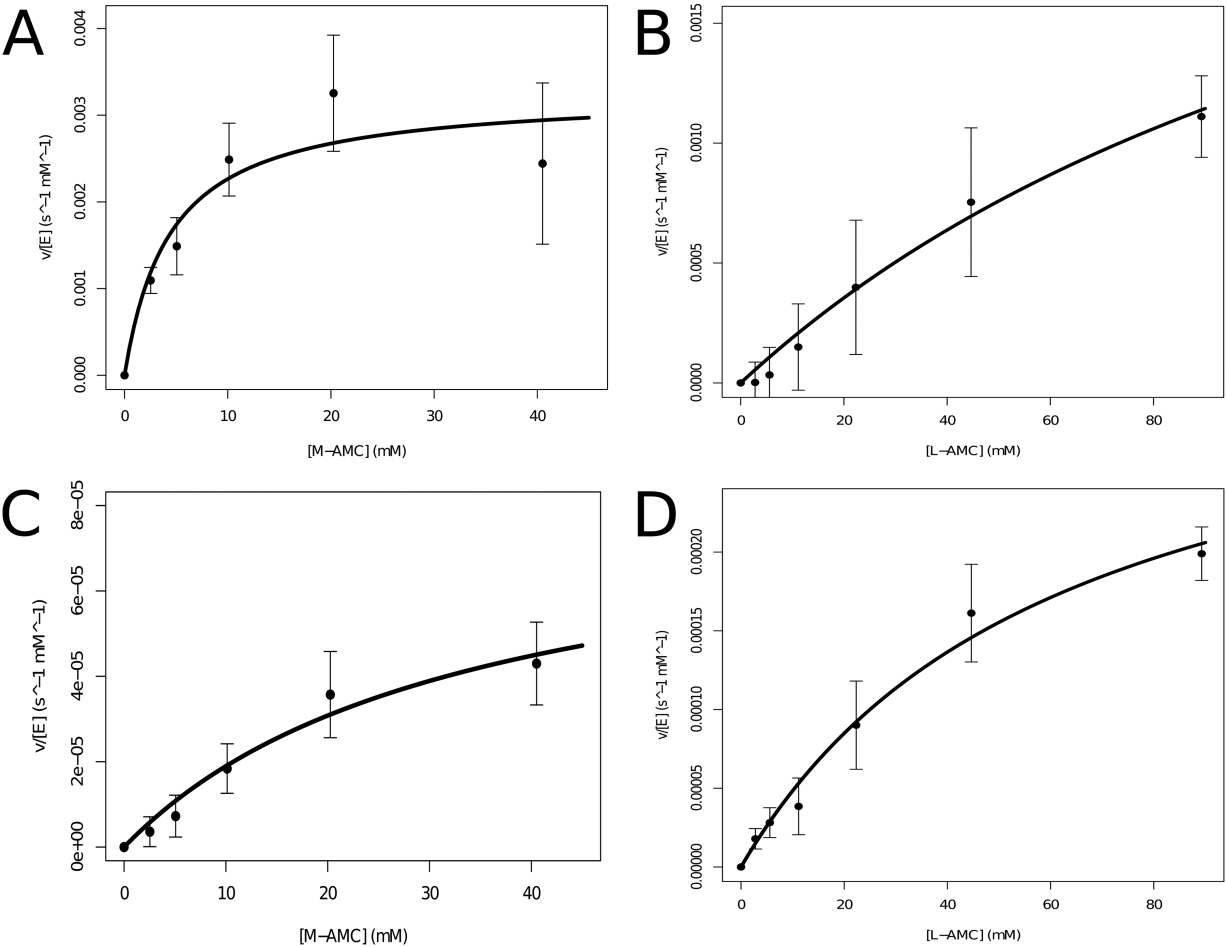
Enzyme kinetics data for (A) eMAP cleaving methionine (eMAP-met), (B) eMAP-leu, (C) eLAP-met, and (D) eLAP-leu. Best-fit curves using the Michaelis-Menten model are overlaid along with error bars for reaction velocities measured in triplicate. Parameters are listed in Table II.

To determine whether the change in binding specificity translates into a change in substrate specificity, we characterized the enzymatic activity of both eMAP and eLAP using a fluorogenic assay. When the substrate [Met/Leu]-AMC is cleaved, the liberated AMC group fluoresces, allowing direct measurement of substrate accumulation. While optimal eMAP activity is known to require longer peptide substrates for maximal activity, we selected these substrates for ease of measurement, and because we are interested in relative, rather than absolute, rates. The Met-AMC substrate is roughly equivalent to a two-amino acid substrate, which according to previous reports should be cleaved with an activity around 5% of that of a pentapeptide.^31^ We measured the activities of both enzymes against both substrates. Initial velocities for each substrate (RFU min^−1^) were converted to concentrations of released AMC, and initial velocities (μ*M* min^−1^) were plotted as a function of substrate concentration. We determined the kinetic constants for both enzymes against Met-AMC and Leu-AMC; the results are summarized in Table II. The measured *k*_cat_/*K*_m_ (catalytic efficiency) for eMAP against the Met-AMC and Leu-AMc substrates was 0.74 s^−1^ *M*^−1^ and 0.02 s^−1^ *M*^−1^, respectively (Fig. 6). Thus, the catalytic efficiency of eMAP against Leu-AMC substrate is ∼2.7% of that against Met-AMC, similar to the relative efficiencies found in a previous study utilizing pentapeptides (∼3%),^31^ indicating that despite the difference in absolute magnitudes, the AMC substrate provides an accurate measurement of relative catalytic efficiency.

**Table II:** Kinetic parameters of native and designed proteins

Protein/substrate	*k*_cat_ (s^−1^)	*K*_m_ (µ*M*)	*k*_cat_/*K*_m_
Emap/Met-AMC	0.0033	0.0044	0.7397
Emap/Leu-AMC	0.0031	0.157	0.0201
Elap/Met-AMC	8.22 × 10^-5^	0.0336	0.0024
Elap/Leu-AMC	3.48 × 10^-4^	0.0619	0.00562

We next assayed the activity of eLAP against both substrates. The engineered enzyme has a *k*_cat_/*K*_m_ of.0056 s^−1^ *M*^−1^ against Leu-AMC, and a *k*_cat_/*K*_m_ of.0024 s^–1^ *M*^–1^ against Met-AMC. While the lower activity indicates that our mutated enzyme is significantly less efficient than the native for both substrates, eLAP is more than twice as efficient at cleaving the Leu-AMC substrate than the Met-AMC. The *K*_m_ of eLAP for the methionine substrate is actually lower than the *K*_m_ for the leucine substrate. However, *k*_cat_ is nearly two orders of magnitude worse. We speculate this is due to the significantly greater conformational heterogeneity of the methionine side-chain and the adoption of a greater variety of bound states, which though they are tightly associated, render catalysis impossible. The notion of a less-ordered loop is consistent with an observed decrease in solubility of eLAP versus eMAP.

## Discussion

The ability to engineer specific activities into proteins using computational techniques has advanced rapidly over the past several years and has enormous potential for generating novel therapeutics, industrial enzymes, and biotechnology tools. Current algorithms rely heavily on harnessing the native properties of existing proteins, either explicitly through the use of rotamers or implicitly through knowledge-based energy terms, to reconstruct enzymes or proteins with altered activity or specificity. In algorithms that explicitly target predefined interactions, stable modules that possess the desired properties are either designed or identified from a database of empirical structures and computationally matched to a region in the target protein. This type of “building-block” approach to synthetic biology has become popular in metabolic engineering^32^–^35^ and several reports have demonstrated its applicability to protein design.^7^,^8^,^36^,^37^

Perhaps the most striking examples of module transplantation have been generated as a product of *de novo* enzyme engineering. The Rosetta software suite's enzyme design protocol, for example, has successfully transplanted artificial active sites onto native backbone scaffolds.^7^,^9^,^38^ While this is an extraordinary feat of protein engineering, and a stringent test of our understanding of protein structure and function, *de novo* design is a much more difficult problem than need be solved to generate novel enzymatic activities for many practical applications. By contrast, redesign of native enzymes requires less effort and can draw upon a supply of over 4000 chemical activities^39^ that could be amenable to redesign.

We note that although computational design with motif-directed backbone flexibility was successful in this case, the two motifs that were incorporated into the flexible region of the protein both involved hydrophobic residues. It remains to be seen whether this approach will prove successful for the design of hydrogen-bonded or electrostatic interactions, which has proven more difficult than design involving only hydrophobic contacts.^40^,^41^ Hydrogen bonded interactions require more stringent geometric constraints, and the many-body, networked nature of these interactions may be a poor fit for standard design schemes, which typically employ scoring functions that are truncated at two-body terms.

In eMAP, the catalytic, metal-chelating residues are readily distinguished from the side-chain specificity-determining residues. Approaches such as motif-directed design are likely to work well in proteins where this is the case, or when the desired function is limited to binding or recognition. In this study, eLAP exhibited binding kinetics for an N-terminal leucine that were similar to those of eMAP for an N-terminal methionine. In general, however, specificity, catalysis, and binding energy are intimately entangled in enzymes.^42^ Our results from the enzymatic assays indicate that in the case of eMAP this is minimal but still apparent, as the *k*_cat_ of the designed eLAP is significantly less than that of the native enzyme for both substrates. This may limit the applicability of the residue level, motif-based approach for the design of certain novel catalysts.

Despite this limitation, our results also suggest that this approach may work well in combination with directed evolution. Directed evolution is very effective at optimizing a pre-existing activity, but is often incapable of generating large, coordinated changes to establish novel function. Our results demonstrate the ability of motif-based computational design to change specificity, and to cope with a large number of mutations (19 for eLAP relative to eMAP). While it is likely that not all mutations are essential for our desired goal, such a large number of simultaneous mutations cannot be encoded in a genetic library. In cases where a preexisting activity is lacking, motif-based design may thus allow protein engineers to generate a starting point that would not be discoverable by directed evolution alone. It is also possible that directed evolution would be able to identify additional mutations to improve upon the activity of eLAP.

## Methods

### Construction of motif library

We extracted motifs from a subset of the PDB^43^ obtained from the PISCES server.^44^,^45^ We required that all structures be solved using x-ray crystallography to a resolution of 1.6 Å or better, with R-factors of 0.25 or better, and that no two domains shared more than 20% sequence identity. This yielded 1682 structures. For each structure, all residue-residue interactions that include a leucine were scored using the Rosetta full-atom scoring function. We isolated hydrophobic interactions by considering individual scoring terms. If the total Lennard-Jones potential score was greater than –1.0 Rosetta energy units (REU) (lower values are more favorable), the interaction was discarded. Otherwise, the geometry between the sidechains was determined as previously described.^19^ Briefly, a coordinate system is defined for each of the amino acids by predefined terminal heavy atoms (e.g., Cγ, Cδ1, and Cδ2 for leucine). The translation vector and rotation matrix relating the coordinate systems between residues is obtained, and may be used to recreate one interacting partner given the other. To eliminate redundant interactions, the geometric transformation is checked against previously calculated examples. Any interaction whose translation vector and rotation matrix differ from another by less than 1.0 Å and 0.4 radians, respectively, are deemed to be redundant and are discarded. Each such interaction (called a motif) is defined by the identities of the amino acids involved, the atoms used to define the coordinate systems, and the transformation relating the two systems. We call the resulting set of nonredundant, previously observed interactions a motif library.

### Design template preparation

The starting point for redesign calculations was the experimentally determined structure for eMAP in complex with the transition state analog norleucine phosponate (PDB code: *2GTX*^46^. We modeled a leucine amino acid superimposed upon the norleucine phosponate, and predicted the favored side chain conformation using the Rosetta program.^47^ The conformation of the leucine amino acid was held fixed for all subsequent calculations. Residues 56–70 were selected as a loop region. In order to give the loop region flexibility in accommodating interactions with the leucine substrate, residues 56–70, as well as neighboring residues 42,46,81,101,177, and 221 were replaced with alanine, with the exception of glycine and proline residues, which were not changed.

### Motif incorporation

The procedure for motif-directed backbone movement and incorporation of interacting virtual amino acids is given in detail in Ref.^19^. Briefly, the leucine-specific motif library was used to generate possible interactions between the protein and the leucine substrate in two steps. First, the geometric information for each motif was used to place a virtual interacting amino acid in contact with the substrate. The motif defines the relative orientation of the terminal heavy atoms in the interacting amino acid. Second, we made copies of each virtual amino acid that differed only in their side chain torsion angles, which were taken from a rotamer library. Copies that clashed with the substrate or residues outside the loop region, or that had main chain atoms too far from any protein residue (rmsd > 2.0 Å over the C_β_, C_α_, C, and N atoms) were discarded. Thus, each motif in the motif library gives rise to multiple virtual amino acids, each satisfying the geometric requirements of the motif interaction, but with different locations for their backbone atoms.

We next determined whether the protein backbone atoms could be made to superimpose with those of each virtual interacting amino acid. We performed loop relaxation under the Rosetta scoring function augmented with harmonic constraints between the C_β_, C_α_, C, and N atoms of the amino acid and the corresponding atoms of the closest backbone position in the flexible loop. Backbone movement was considered successful if the final rmsd over the constrained atoms was below 1.0 Å. In this case, the virtual motif amino acid was modeled onto the backbone. As aligning the backbone atoms causes the terminal atoms to shift, we performed a second round of loop relaxation in which constraints are applied to restore the motif-defining terminal atoms to their ideal locations. We accepted as successful those cases with final rmsd values below 1.0 Å. These motif-incorporating models served as the starting point for further attempts to incorporate additional motifs.

### Redesign of loop residues

Following the final motif placement, surrounding residues in the design region are mutated to support the altered backbone conformation using a combination of standard protocols for fixed backbone design and energy minimization from RosettaDesign.^28^ First, we redesigned any positions in the flexible loop or the neighboring residues that had been replaced with alanine prior to motif incorporation, excluding incorporated motif residues. Then, the “backrub” loop relaxation protocol was applied to the loop (residues 56–70).^30^ We performed 10 iterations of this combined procedure.

### Cloning and mutagenesis

The gene for methionine aminopeptidase was amplified from *E. coli* genomic DNA, cloned into the pET42(a) expression vector (Novagen, MA) upstream of a 6× his-tag, and verified by sequencing. Site directed mutagenesis was done by the Kunkel method, with oligos ordered from IDT and mutants verified by sequencing. Proteins were expressed using an autoinduction protocol.^48^ Proteins were purified by immobilized metal affinity chromatography, eluted with an imidazole gradient, and concentrated by ultrafiltration. Identity and purity were verified by SDS-PAGE. Purified protein was dialyzed against 1x phosphate buffered saline (pH 7.4) for 24 h, and stored in 50% glycerol. Concentrations were determined by absorbance at 280 nm.

### Binding assays

Substrate peptides with a sequence of X-GMMSC were obtained (Cel-Tek, TN), where X is either methionine or leucine. Biolayer interferometry using the BLItz platform (Forte Bio, CA) requires the immobilization of the substrate onto a fiber optic tip coated with amine-reactive chemical groups. Each substrate was attached by activating the tip with a 0.4*M* 1-ethyl-3-3-dimethylaminopropyl)-carbodiimide (EDC) and 0.1*M N*-hydroxysuccinimide (NHS) solution for 10 min, attaching a *N*-beta-maleimidopropionic acid hydrazide (BMPH) hetero-bifunctional crosslinker in 0.1*M* sodium borate at pH 8.5 to introduce an exposed, reactive thiol, quenching unreacted amine-reactive groups with 1*M* ethanolamine, attaching the substrate by reacting the C-terminal Cysteine with the BMPH thiol, and quenching unreacted thiols with a solution of 50 m*M* cysteine and 1*M* NaCl in 0.1*M* sodium acetate at pH 4.3. The substrate derived tips where then washed with 100 μ*M* BSA and stripped to remove any protein contaminants with 8*M* guanidine chloride twice before starting the binding assay. To measure affinity constants, each tip was first blanked against a 1x PBS buffer containing no protein. 4 uL of 1x PBS containing 3.7 μ*M* (eMAP) or 4.9 μ*M* (eLAP) were loaded and the association of the protein to the subtrate was measured for 2 min. The tip was transferred back into a 1x PBS blank and dissociation kinetics were measured for 2 min. The tip surface was then washed with 8*M* guanidine chloride to strip off any remaining protein before the tip was reused. Negative controls with both BSA and the buffer blank showed no association/dissociation curves. Data was globally fit using the built-in BLItz software to a 1:1 binding model to determine *k*_on_, *k*_off_, and *K*_D_.

### Aminopeptidase assays

Fluorogenic amino-methylcoumarin substrate (x-AMC, where x is either methionine or leucine) were ordered from BaChem. Cleavage of AMC from the substrates was monitored on a 96-well plate fluorometer using a Synergy 2 Multi-Mode Microplate Reader (Bio-Tek, VT) at an excitation wavelength 360 nm and an emission wavelength 485 nm for all substrates. Assays were conducted on 96-well round bottom black polystyrene microplates (Corning Life Sciences, MA) in a reaction volume of 150 µL containing 3.7 μ*M* eMAP or 4.9 μ*M* eLAP, assay buffer (1× PBS, pH 7.4) and substrate at concentrations ranging from 1 to 80 m*M*. Reaction mixtures were held at 4 C during combining, preincubated for 1 h at 25°C and started by addition of 10 μ*M* cobalt chloride to the mixture. Fluorescence accumulation was monitored every 1 min over a period of 60 min and relative fluorescence units were converted to rates of substrate cleavage by calibration with a free AMC standard curve (Sigma Aldrich, MO). Reaction rates at steady state were calculated from the slope of the fluorescence time courses by linear regression of initial velocities, and kinetic parameters were calculated assuming Michaelis-Menten kinetics, *v* = *V*_max_(*S*)/(*S*) + *K*_m_ by nonlinear regression in the *R* statistical software package.
